# The Prevalence of von Willebrand Disease and Significance of in Vitro Bleeding Time (PFA-100) in von Willebrand Disease Screening in the İzmir Region

**DOI:** 10.4274/tjh.2011.0020

**Published:** 2013-03-05

**Authors:** Fatih Şap, Tülay Kavaklı, Kaan Kavaklı, Ceyhun Dizdarer

**Affiliations:** 1 Konya Training and Research Hospital, Department of Pediatrics, Konya, Turkey; 2 Dr. Behçet Uz Children’s Research and Training Hospital, Department of Pediatrics, İzmir, Turkey; 3 Ege University Faculty of Medicine, Department of Pediatric Hematology, İzmir, Turkey; 4 Dr. Behçet Uz Children’s Research and Training Hospital, Department of Pediatric Endocrinology, İzmir, Turkey

**Keywords:** von Willebrand disease, prevalence, PFA-100, sensitivity, Specificity

## Abstract

**Objective:** von Willebrand disease (vWD) is the most common hereditary bleeding disorder. The purpose of this investigation was to determine the prevalence of vWD among adolescents in İzmir and to assess the sensitivity and specificity of PFA-100 as a screening method in detecting this disease.

**Material and Methods:** Our study was conducted on adolescents in the city of İzmir between October 2006 and March 2007. A total of approximately 1500 high school students between 14 and 19 years of age were planned to be included in the investigation. Survey forms prepared for assessing hemorrhagic diathesis were completed by 1339 individuals (512 males, 827 females). The necessary laboratory tests were performed after having obtained written informed consent from 40 individuals suspected to have hemorrhagic diathesis.

**Results:** Based on the von Willebrand factor antigen (vWF:Ag) and ristocetin cofactor activity (vWF:RCo) levels and bleeding symptoms, vWD type-1 was diagnosed in 14 individuals (4 males, 10 females; prevalence: 1.04%). The most common bleeding symptom in these patients was found to be epistaxis (10/14). Screening with PFA-100 revealed prolongation in both cartridges (Col/ADP and Col/Epi) in 3 of the 14 patients. PFA-100 was determined to exhibit 21.4% sensitivity and 100% specificity in the diagnosis of vWD.

**Conclusion:** The PFA-100 device was found to have high specificity but to have exhibited low sensitivity. Therefore, its utilization as a screening test may be problematic in patients with mild type-1 vWD. Specific tests (vWF:RCo, vWF:Ag) are required for the definite diagnosis of vWD. However, further studies with a large number of patients are needed.

**Conflict of interest:**None declared.

## Abstract (tr)

**Amaç:** von Willebrand hastalığı (vWH) en sık kalıtsal kanama bozukluğudur. Bu çalışmanın amacı, İzmir’de adolesanlarda vWH’nın sıklığını saptamak ve tarama yöntemi olarak PFA-100’ün bu hastalığı saptamada duyarlılık ve özgüllüğünü belirlemektir.

**Gereç ve Yöntemler:** Çalışmamız İzmir İli’nde adolesanlarda Ekim 2006-Mart 2007 tarihleri arasında yapıldı. Yaşları 14-19 arasında olan yaklaşık 1500 lise öğrencisinin çalışmaya dahil edilmesi planlandı. Kanama diyatezini sorgulamaya yönelik hazırlanan anket formlarının cevapları 1339 (512 erkek, 827 kız) bireyden geri toplanabildi. Muhtemel kanama diyatezi olduğu düşünülen 40 bireyden yazılı onam formları alınarak gerekli laboratuvar testleri alındı.

**Bulgular:** von Willebrand faktör antijen (vWF:Ag) ve ristosetin kofaktör aktivite (vWF:RCo) düzeyi ve kanama semptomları esas alınarak 14 bireyde (4 erkek, 10 kız) von Willebrand Hastalığı tip-1 tespit edildi (prevalans %1,04). Bu hastalarda saptanan en sık kanama semptomunun burun kanaması olduğu (10/14) görüldü. PFA-100 ile yapılan taramada ise 14 hastadan 3’ünde iki kartuşta da (Col/ADP ve Col/Epi) uzama görüldü. PFA-100’ün von Willebrand Hastalığı’nı saptamadaki duyarlılığı %21,4 ve özgüllüğü %100 olarak bulundu.

**Sonuç:** Sonuç olarak PFA-100 cihazı yüksek oranda özgül olmakla birlikte düşük oranda duyarlı bulunmuştur. Hafif tip-1 vWH için tarama testi olarak kullanılması sorun yaratabilir. von Willebrand hastalığının mutlak tanısı için spesifik testler (vWF:RCo, vWF:Ag) gerekmektedir. Ancak daha fazla hastayı içeren ileri çalışmalara ihtiyaç vardır.

## INTRODUCTION

von Willebrand disease (vWD) is an autosomal hereditary bleeding disorder associated with a quantitative or qualitative defect of von Willebrand factor (vWF) [1]. The vWF is responsible for transport and stabilization of factor VIII (FVIII), in addition to provision of adhesion of platelets to the endothelium. A serious deficiency or a structural defect of vWF leads to secondary FVIII deficiency and causes bleeding diathesis [1,2]. The disease is classified as type-1 in cases with a decreased amount of vWF (quantitative deficiency), as type-2 (qualitative defect) in cases with a structural defect of vWF, and as type-3 in cases with no vWF protein or function [1,2]. Type-1 vWD, the most common form of the disease, is characterized by quantitative deficiency of vWF and presence of mild bleeding episodes in the patient and also in family member [3].

vWD is the most common hereditary bleeding disorder and its incidence in the community has been reported as 1%-2% in western literature [2]. It was first described in 1926 by Dr. Eric von Willebrand from Finland as a disease differentiated from hemophilia based on the clinical findings [4]. In 3 separate investigations conducted in Turkey, the prevalence was found to be 0.7% in the Ankara region [5], 0.44% in the İzmir region [6], and 0.9% in the Edirne region [7]. 

The history is critical in the diagnosis of the disease and most patients present with mucocutaneous bleeding symptoms. Persistent bruises, epistaxis, mucosal bleeding, prolonged bleeding following tonsillectomy and tooth extractions, prolonged menstrual bleeding, and spontaneous gastrointestinal system bleedings are indicative of this disease [1,2,8,9]. 

Determination of a decrease in ristocetin cofactor activity (vWF:RCo), clinical symptoms related to bleeding, and family history are diagnostic for vWD. In type-3 vWD, prolonged bleeding time and prolonged activated partial thromboplastin time (aPTT) are typical. On the other hand, these tests are usually normal in type-1 vWD. Therefore, it is not possible to exclude the diagnosis of vWD with normal screening test results [2]. 

It is possible to evaluate the bleeding time, both in vitro and in vivo. The in vivo method is outdated, difficult to apply, and less sensitive when compared to the in vitro method (PFA-100). The PFA-100 (Platelet Function Analyzer) device measures the bleeding time based on the closure time [10,11]. This test is a useful screening method for evaluation of vWD and various acquired or congenital intrinsic platelet function disorders. Since the in vitro method is less invasive, is repeatable, has a certain standard for interpretation of results, and is superior to the in vivo method in terms of sensitivity and specificity, it is the preferred method in practice [10]. 

In this study, our purpose was to determine the prevalence of vWD among adolescents in the city of İzmir and to evaluate the sensitivity and specificity of PFA-100 as a screening method for this disease.

## MATERIALS AND METHODS

This investigation was conducted among adolescents attending 3 different high schools in the Konak District of İzmir during the period between October 2006 and March 2007. Approximately 1500 high school students between 14 and 19 years of age were planned to be enrolled. A survey form (Supplement 1) [6] containing 8 questions was distributed to be completed by students and their families. With the survey form, the presence of bleeding symptoms and quality, frequency, and severity of these symptoms (if any) were investigated among the adolescents and their families. A total of 1339 survey forms were completed and returned and these were evaluated by a specialist physician experienced in the field of pediatric hematology. Acceptance of significant bleeding symptoms in terms of vWD was designated according to the criteria recommended by the International Thrombosis and Hemostasis Society (ISTH). The diagnosis of patients with vWD was established with decrement of vWF:RCo and/or vWF antigen (vWF:Ag), presence of meaningful bleeding symptoms, and also similar symptoms in at least one family member according to ISTH criteria [3]. In 40 individuals suspected to have hemorrhagic diathesis (9 males, 22.5%; 31 females, 77.5%), blood samples were obtained for further evaluations. The blood samples were examined for complete blood count (Sysmex XT-2000i hemocounter, Japan) and prothrombin time (PT)/aPTT/fibrinogen (STA Compact, Diagnostica Stago, France) values in the hematology laboratory of Dr. Behçet Uz Children’s Hospital; the samples of individuals with no approved certificates of blood group were examined in the blood center of Dr. Behçet Uz Children’s Hospital. PFA-100 (In vitro Bleeding Time, Dade Behring, Germany), vWF:Ag (Sysmex CA-1500), and vWF:RCo (Biodata platelet aggregation device, USA) levels were evaluated at the hemostasis laboratory of Ege University Medical School’s Department of Pediatric Hematology. The normal lower limit value of vWF:Ag was accepted as 65% and the normal value for vWF:RCo was accepted as over 50%, but for individuals with the O blood group, the lower limit for vWF:RCo was considered as 45%. Our laboratory normal reference ranges in healthy controls and also data of the laboratory company indicated these ranges. Recent studies revealed that people with blood group O normally had lower levels in vWF tests than non-O individuals [12,13]. In a prior study, the closure time was determined as 71-176 s for collagen epinephrine closure time (Col/Epi) and 70-169 s for collagen adenosine diphosphate closure time (Col/ADP) [10]. However, we used our laboratory normal reference ranges, such as 85-165 s for Col/Epi and 71-118 s for Col/ADP. 

The diagnosis of vWD was confirmed by bleeding symptoms, in addition to low levels of vWF:RCo and/or vWF:Ag and presence of a family history of bleeding. Furthermore, PFA-100 screening was performed in patients suspected to have hemorrhagic diathesis and the sensitivity and specificity of this method were investigated among vWD patients. 

The study was commenced after having obtained approval from the local ethics committee and appropriate written consent from the İzmir City Board of Health and Ministry of Education. Informed consent forms were obtained from the parents of individuals subjected to blood sample analysis.

Statistics: In this investigation, the Student t-test, the Fisher exact test, and the Pearson correlation and chi-square tests were utilized. P-values of less than 0.05 were regarded as significant; in the evaluation of correlations, negative or positive correlation rates among values with a significant P-value were calculated.

## RESULTS

The survey forms were distributed among 1400 students, but the number of completed and returned forms was 1339 (512 males, 38.2%; 827 females, 61.8%). Among these students, 115 individuals were regarded as significant in terms of hemorrhagic diathesis. Upon reevaluation of the symptoms, 46 students were considered as having insignificant symptoms and these individuals were excluded from the study; no contact was possible with 20 students. Among the remaining 49 individuals, 40 students (9 males, 31 females) gave consent for further blood tests; hence, blood samples were obtained from these individuals. The mean age of the 40 individuals was 15.9 years (minimum: 14 years, maximum: 19 years, SD: ±1.16). No thrombocytopenia was encountered in any of the cases, but mild anemia was present in 5 individuals. Furthermore, PT, aPTT, and fibrinogen levels were determined to be within normal limits.

In 2 individuals, both vWF:Ag and vWF:RCo levels were found to be low, and in 12 individuals, only the vWF:RCo level was determined to be decreased. In 2 students, vWF:RCo exhibited values close to limits like 45%, but evaluation of the blood groups revealed no individuals with blood group O. In vWD, the vWF:RCo value is regarded as the gold standard diagnostic method; therefore, 14 of the 40 suspected patient (4 males, 10 females) were determined to have type-1 vWD. No significant difference was found between the group diagnosed as vWD (14 individuals) and individuals with no disease (26 individuals) according to the results of the blood tests, in terms of age and gender distribution (p=0.77, p=0.70, respectively). 

Upon evaluation of the bleeding symptoms of 14 cases of vWD, the most frequent symptom was epistaxis in 10 patients (71%). The following symptoms were found in addition to epistaxis: easy bruising of skin in 2 of our patients (14%), prolonged bleeding following mucocutaneous surgery in 1 patient (7%), more than 7 days of menstrual bleeding in 2 of the 10 female patients (20%), prolonged bleeding in superficial lacerations in 6 patients (42%), and prolonged bleeding following tooth extraction in 2 patients (14%). In our male patients, no history of prolonged bleeding was found following circumcision. Serious or deep (intramuscular, intraarticular, intracranial, etc.) bleeding symptoms were not observed in any of our patients. The bleeding symptoms and PFA-100 and vWF levels of the cases of vWD are presented in Table 1. Upon evaluation of all bleeding symptoms in our patients and comparison in terms of the presence of similar symptoms in the family history, no significant difference was found between vWD patients and the group with no disease (p>0.05). 

Based on vWF:Ag and vWF:RCo, 14 of the 40 suspected cases were diagnosed as type-1 vWD. Hence, the prevalence of type-1 vWD was determined as 1.04% (14/1339). The PFA-100 test was performed with 2 separate cartridges containing epinephrine and ADP. In the PFA-100 analysis, prolongation in both cartridges (Col/Epi-Col/ADP) was observed in 3 patients who had been diagnosed with type-1 vWD. In another group of 3 cases, single prolongation was detected in the in vitro bleeding time (Col/Epi). In 2 of these 3 individuals with single prolongation (Col/Epi), administration of a drug with an effect of prolongation of bleeding time was present; the other patient with single prolongation (Col/Epi) was diagnosed with type-1 vWD. When dual prolongation was accepted as significant, including individuals who had received drugs causing prolonged bleeding time, the sensitivity was 21.4%, the specificity was 100%, the positive prediction value (PV+) was 100%, and the negative prediction value (PV−) was 70.3% at a 95% confidence interval; when individuals receiving drugs were excluded, the sensitivity, specificity, and PV+ values did not change at the 95% confidence interval, but the PV− was determined as 67.6% (Table 2). 

Comparison of the group diagnosed with disease (n = 14) and the group with no disease (n = 26) with a history of hemorrhagic diathesis revealed significantly different values for vWF:Ag, vWF:RCo, Col/Epi, and Col/ADP (p<0.05) (Table 3).

## DISCUSSION

In the past, vWD was generally not well known in Turkey. There are still some problems in respect to laboratory diagnosis and classification, in spite of the fact that awareness of the disease has increased lately. However, the incidence of vWD is quite high (1%-2%) [2]. Some recent articles have indicated that evaluation by in vitro bleeding time (PFA-100) is logical, since vWD plays a role in the primary hemostasis mechanism [14,15,16]. This test is argued to be more sensitive when compared to the in vivo (Ivy) method [10,14,17,18,19]. 

Various investigations have been conducted on the prevalence of vWD in Turkey and around the world. Rodeghiero et al. [20] and Werner et al. [21] determined the prevalence of vWD as 0.82% and 1.3%, respectively. In Turkey, 3 separate prevalence investigations were conducted in the Ankara, İzmir, and Edirne regions and the prevalences were found to be 0.7%, 0.44%, and 0.9%, respectively [5,6,7]. 

Since vWF is an acute phase reactant, the possibility of false negative results during stress or infection has been considered an issue. Low vWF activity was determined in 14 patients (10 females, 4 males). Among these individuals, vWF:Ag was <65% in only 2 of the cases. Furthermore, 2 patients with vWF:RCo values closer to the limits had blood groups other than O. Since we were unable to determine the blood group in some cases, no comparison was performed between O and other blood groups in terms of vWF levels. Since vWD patients diagnosed in our investigation were individuals with no serious bleeding symptoms, the diagnosis in all of our cases was confirmed as mild type-1 vWD. Therefore, the prevalence of vWD based on our results was determined as 1.04% (14/1339). In a recent study, the ratio of vWF:RCo to vWF:Ag was found to be unreliable in differentiating severe type-1 vWD from type-2 vWD [22]. Therefore, this parameter was not used in our study. 

The most common symptom observed among our patients was epistaxis (71.4%). Similarly, in previous studies conducted in İzmir and Edirne, epistaxis was again the leading symptom [6,7]. The second most frequent symptom in our cases was prolonged bleeding following superficial abrasions. In previous studies [6,7], the second most common symptom was determined as easy bruising. The fact that these investigations were conducted on primary school children, who are frequently exposed to trauma, explains the cause of easy bruising. However, Sidonio et al. [23] reported that a personal or family bleeding history at presentation and the presence of 2 or more bleeding symptoms were not found to be predictive of vWD, low vWF, or nonspecific defective platelet aggregation. They also revealed that qualitative assessment of bleeding symptoms alone was not worthwhile in children. In contrast, Tosetto et al. [24] reported that the usage of quantitative bleeding assessment tools contributes to the evaluation of patients with suspected mild bleeding disorders. 

Although PFA-100 is more sensitive in determining the serious types of vWD (severe type-1, type-2, and type-3), it has been found to be prolonged in a number of type-1 vWD patients. Nevertheless, it may be found to be normal in mild type-1 vWD cases, or prolongation may be seen in the Col/Epi cartridge only [14,17]. In our study, prolongation was determined in the Col/Epi cartridge in 6 patients, and in 3 of these individuals, prolongation was also observed in the Col/ADP cartridge. In other words, dual prolongation was present in 3 patients and these individuals were diagnosed with type-1 vWD. Therefore, the sensitivity of PFA-100 in diagnosing vWD was determined as 21.4% (3/14). When dual prolongation is accepted as significant, including individuals who had received drugs causing prolonged bleeding time, the sensitivity was 21.4%, the specificity was 100%, the PV+ was 100%, and the PV− was 70.3% at a 95% confidence interval; when individuals receiving drugs were excluded, the sensitivity, specificity, and PV+ values did not change at the 95% confidence interval, but the PV− was determined as 67.6%. With these results, our false positive rates were zero, but the false negative rates were found to be high (78.5%; 11/14). According to these values, the PFA-100 seems to be insufficient in screening for mildly symptomatic vWD, and the sensitivity value was much lower than expected when compared to other studies [10,12,14,15,17,18,19,25]. Additionally, in an unselected population, authors revealed PFA-100 to be useful to exclude vWD; however they could not be sure whether it might replace the specific vWF tests in patients with significant mucocutaneous bleeding symptoms [26]. 

Comparison of the group diagnosed with disease and the group with no disease among 40 cases with a history of hemorrhagic diathesis revealed significantly different values for vWF:Ag, vWF:RCo, Col/Epi, and Col/ADP. In other words, although most of the individuals had normal PFA-100 values in spite of being diagnosed as vWD patients, the values were found to be prolonged when compared to the group with no disease. 

In conclusion, the prevalence of vWD among adolescents in the Aegean region of Turkey was determined as 1.04%. The in vitro bleeding time (PFA-100) test can be considered a worthwhile addition to any hemostasis laboratory involved in vWD investigation. The low sensitivity of this screening method may lead to the overlooking of certain patients with mild type-1 vWD. However, severe forms of vWD (severe type-1, type-2, and type-3) can be easily detected with PFA-100. Therefore, specific vWF tests (vWF:RCo, vWF:Ag) are definitely required for confirmation of the diagnosis in patients with mildly symptomatic vWD. However, the number of patients in the present study was low to give an exact conclusion for the PFA-100 test. Further studies including more patients are needed. 

**Conflict of Interest Statement**

The authors of this paper have no conflicts of interest, including specific financial interests, relationships, and/ or affiliations relevant to the subject matter or materials included.

## Figures and Tables

**Supplement 1:**
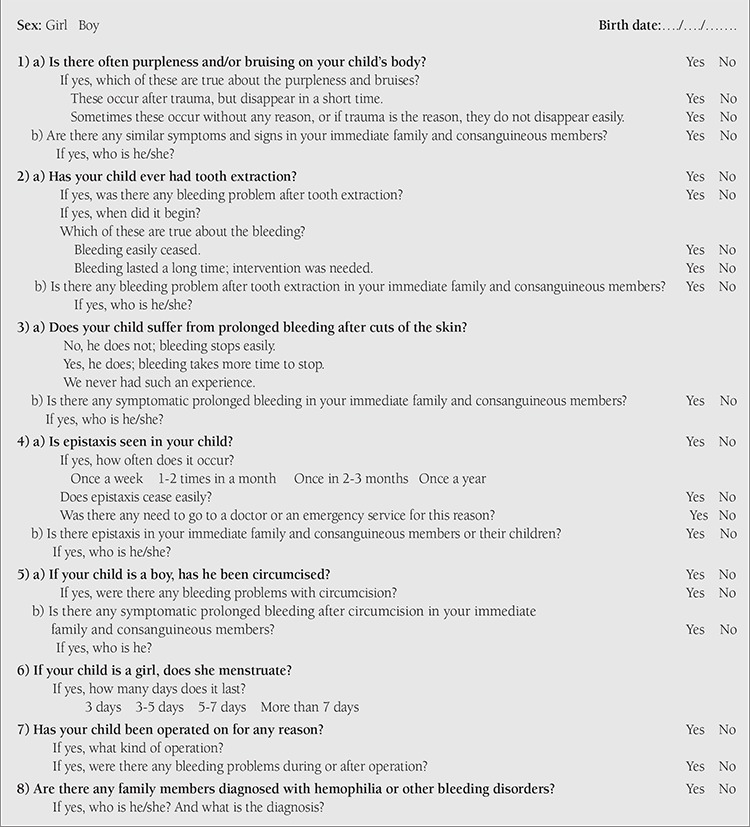
Bleeding tendency questionnaire form [taken from reference 6].

**Table 1:**
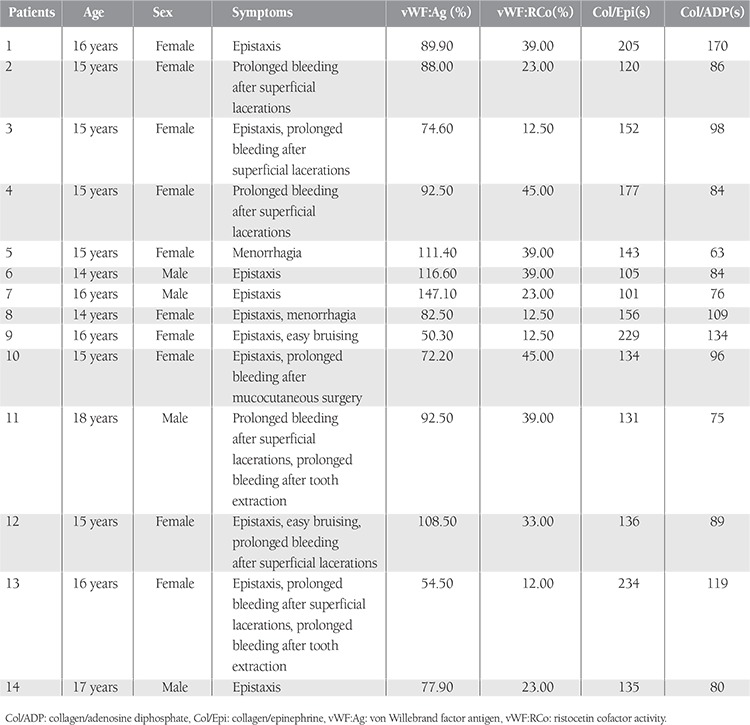
Laboratory and clinical findings of the patients diagnosed with type-1 von Willebrand disease.

**Table 2:**
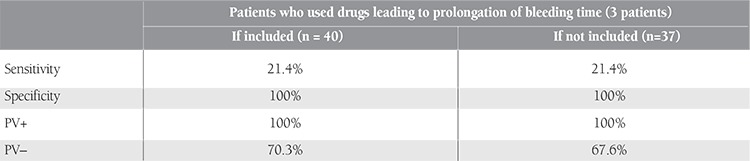
Sensitivity, specificity, and predictive values of PFA-100 to detect vWD with both cartridges (Col/Epi & Col/ADP).

**Table 3:**
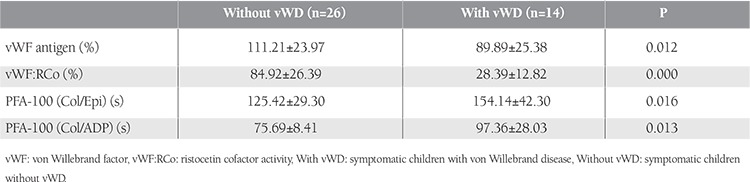
von Willebrand factor and in vitro bleeding time values of all symptomatic children.
